# Harnessing the TAF1 Acetyltransferase for Targeted Acetylation of the Tumor Suppressor p53

**DOI:** 10.1002/advs.202413377

**Published:** 2024-12-24

**Authors:** Md Kabir, Xiaoping Hu, Tiphaine C. Martin, Dmitry Pokushalov, Yong Joon Kim, Yiyang Chen, Yue Zhong, Qiong Wu, Jerry E. Chipuk, Yi Shi, Yan Xiong, Wei Gu, Ramon E. Parsons, Jian Jin

**Affiliations:** ^1^ Mount Sinai Center for Therapeutics Discovery Icahn School of Medicine at Mount Sinai New York NY 10029 USA; ^2^ Department of Pharmacological Sciences Icahn School of Medicine at Mount Sinai New York NY 10029 USA; ^3^ Department of Oncological Sciences Tisch Cancer Institute Icahn School of Medicine at Mount Sinai New York NY 10029 USA; ^4^ Institute for Cancer Genetics and Department of Pathology and Cell Biology and Herbert Irving Comprehensive Cancer Center Vagelos College of Physicians & Surgeons Columbia University New York NY 10032 USA

**Keywords:** acetac, p53y220c, TAF1, targeted protein acetylation

## Abstract

Pharmacological reactivation of the tumor suppressor p53 remains a key challenge for the treatment of cancer. Acetylation Targeting Chimera (AceTAC), a novel technology is previously reported that hijacks lysine acetyltransferases p300/CBP to acetylate the p53Y220C mutant. However, p300/CBP are the only acetyltransferases harnessed for AceTAC development to date. In this study, it is demonstrated for the first time that the TAF1 acetyltransferase can be recruited to acetylate p53Y220C. A novel TAF1‐recruiting AceTAC, MS172 is discovered, which effectively acetylates p53Y220C lysine 382 in a concentration‐, time‐ and TAF1‐dependent manner via inducing the ternary complex formation between p53Y220C and TAF1. Notably, MS172 suppresses the proliferation in multiple p53Y220C‐harboring cancer cell lines more potently than the previously reported p300/CBP‐recruiting p53Y220C AceTAC MS78 with little toxicity in p53 WT and normal cells. Additionally, MS172 is bioavailable in mice and suitable for in vivo efficacy studies. Lastly, novel upregulation of metallothionine proteins by MS172‐induced p53Y220C acetylation is discovered using RNA‐seq and RT‐qPCR studies. This work demonstrates that TAF1 can be harnessed for AceTAC development and expands the very limited repertoire of the acetyltransferases that can be leveraged for developing AceTACs, thus advancing the targeted protein acetylation field.

## Graphical abstract

A lysine acetyltransferase (KAT); can be harnessed for inducing targeted protein acetylation; In this study; it is demonstrated for the first time that TATA‐binding protein‐associated factor 1 (TAF1).

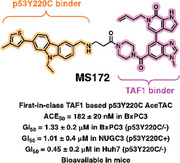

## 1 Introduction

Protein acetylation is one of the most important post‐translational modifications (PTMs) that regulate fundamental biological functions such as cell cycle and chromatin remodeling in cells.^[^ ^1^ ^]^ Lysine acetylation involves the lysine acetyltransferase (KAT)‐catalyzed transfer of the acetyl group from the cofactor acetyl‐CoA to the ε‐amino group of lysine residues, leading to the regulation of distinct protein activity and functions.^[^ ^2^ ^]^ A number of KATs have been identified and they can share substrate specificity and be divided into three main families: 1) GCN5, 2) p300, and 3) MYST.^[^ ^3^ ^]^ On the other hand, lysine acetylation can be reversed by histone deacetylases (HDACs), which include Zn^2+^‐dependent deacetylases and NAD^+^‐dependent deacetylases.^[^ ^3^ ^]^ Dysregulated protein lysine acetylation can lead to the pathogenesis of multiple diseases such as cancer, inflammation and neurodegenerative disorders.^[^ ^4^ ^]^ Thus, the development of a selective and targeted protein acetylation (TPA) strategy is a potential therapeutic approach to thwart the progression of diseases such as cancer.

Previously, we reported a novel technology and modality, termed Acetylation Targeting Chimera (AceTAC), for inducing targeted protein acetylation.^[^ ^5^ ^]^ AceTAC is a heterobifunctional small molecule that induces selective acetylation of a protein‐of‐interest (POI) by hijacking the p300/CBP acetyltransferases.^[^ ^5^ ^]^ We discovered MS78, the first‐in‐class AceTAC, which links a selective small‐molecule binder of p53Y220C to a selective small‐molecule ligand of p300/CBP.^[^ ^5^ ^]^ MS78 induced p53Y220C acetylation in a concentration‐ and time‐dependent manner and had superior antiproliferation activities compared to the parent p53Y220C binder.^[^ ^5^ ^]^ Importantly, AceTAC is the first small molecule‐based TPA modality that does not require any genetic manipulation to induce protein acetylation.^[^ ^6^ ^]^ We also performed structure‐activity relationship (SAR) studies on p300/CBP‐recruiting p53Y220C AceTACs.^[^ ^7^ ^]^ Recently, it was reported that PCAF/GCN5 can be hijacked for protein acetylation using a chemogenetic approach.^[^ ^8^ ^]^ However, this approach requires the genetic tagging of specific proteins,^[^ ^8^ ^]^ making it difficult to develop into cancer therapies.

In this study, we demonstrated that TATA‐binding protein‐associated factor 1 (TAF1), which is the biggest subunit of the multi‐protein Transcription Factor II D (TFIID) complex, can be utilized as a KAT for targeted protein acetylation.^[^ ^9^, ^10^ ^]^ TAF1 is known to initiate the transcriptional preinitiation complex (PIC) formation through the recognition of the core promoter region of genes and also interacts with MYC to regulate gene transcription.^[^ ^11^, ^12^ ^]^ Full‐length TAF1 possesses six distinct domains including an N‐terminal kinase domain, an acetyltransferase domain, followed by ubiquitin‐activating/conjugating domain (E1/E2), two tandem bromodomains categorized as TAF1‐BD1 and TAF1‐BD2, and a C‐terminal kinase domain.^[^ ^10^, ^13^ ^]^ However, whether TAF1 can be hijacked for targeted protein acetylation has never been shown. We discovered the first‐in‐class TAF1‐recruiting p53Y220C AceTAC, MS172, which effectively acetylates p53Y220C without changing p53 protein levels. We validated the MS172‐induced ternary complex formation between p53Y220C and TAF1 via both biochemical and cellular assays. Furthermore, we demonstrated that MS172 is selective for TAF1‐BD2 over several other bromodomain‐containing acetyltransferases. MS172 also exhibits a superior anti‐proliferative effect compared to the parent p53Y220C binder, TAF1 bromodomain binder and p300/CBP‐recruiting AceTAC, MS78, in multiple cancer cell lines harboring the p53Y220C mutation. Lastly, we discovered that the p53Y220C acetylation induced by MS172 upregulates metallothionine protein functions using RNA‐seq and RT‐qPCR studies. Overall, we showed that the TAF1 acetyltransferase can be harnessed for inducing targeted protein acetylation.

## 2 Results and Discussion

### 2.1 Design and Synthesis of TAF1‐Recruiting p53Y220C‐Targeting AceTACs

**Figure 1:**
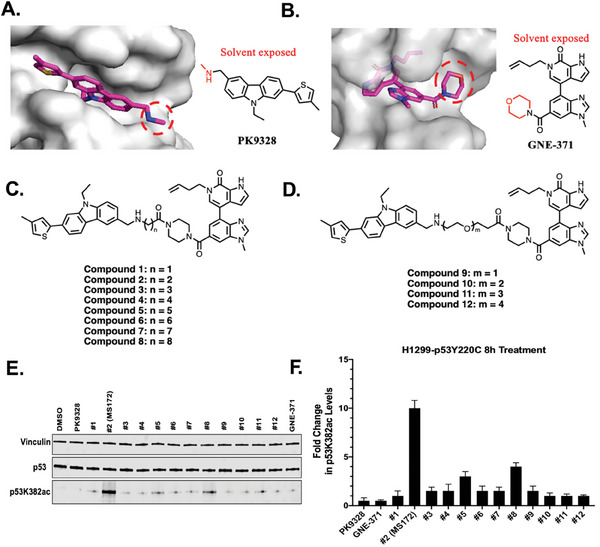
Design and evaluation of TAF1‐recruiting p53Y220C‐targeting AceTACs. A) Co‐crystal structure of the p53Y220C‐PK9328 complex (PDB ID: 6GGF).^[^ ^14^ ^]^ Left: the cross‐section of the p53Y220C binding pocket (in gray) occupied by PK9328 (in magenta). The solvent‐exposed region of PK9328 is highlighted by the red dashed cycle. Right: the chemical structure of PK9328. B) Co‐crystal structure of the TAF1 (BD2)‐GNE‐371 complex (PDB ID: 6DF7).^[^ ^19^ ^]^ Left: the cross‐section of the TAF1‐BD2 binding pocket (in gray) occupied by GNE‐371 (in magenta). The solvent‐exposed region of GNE‐371 is highlighted by the red dashed cycle. Right: the chemical structure of GNE‐371. C) Chemical structures of alkylene linker‐based p53Y220C AceTAC compounds 1–8. D) Chemical structures of PEG linker‐based p53Y220C AceTAC compounds 9–12. E) Representative western blot (WB) results of PK9328, compounds 1–12, and GNE‐371 in H1299‐ p53Y220C stable cells treated with the indicated compound at 5 µm for 8 h (from two independent experiments). Total cell lysate was used for WB and vinculin was used as a loading control. F) Quantification of the fold change of the p53K382ac level (p53K382ac level over total p53 protein level) for the WB results shown in panel E and its biological repeat.

Similar to our previous AceTAC design, we analyzed the co‐crystal structure of p53Y220C in complex with PK9328 (PDB ID: 6GGF), a previously reported p53Y220C small‐molecule stabilizer, and utilized the solvent‐exposed methylamine group as a suitable exit vector for attaching linkers (**Figure** **1**a).^[^ ^5^, ^7^, ^14^ ^]^ To recruit TAF1 for inducing acetylation without significant loss of its KAT activity, we chose to utilize a small‐molecule binder of the TAF1 bromodomain, but not small‐molecule inhibitors of TAF1 acetyltransferase.^[^ ^15^ ^]^ There are several reported TAF1 bromodomain binders including benzoisoquinolinedione‐based compounds, such as BAY‐299,^[^ ^16^ ^]^ naphthyridones, such as compound 23,^[^ ^17^ ^]^ and dual TAF1 and ataxia telangiectasia and Rad3 related (ATR) kinase inhibitor, AZD6738.^[^ ^18^ ^]^ In this study, we utilized GNE‐371, which contains a pyrrolopyridone core and is a potent and selective TAF1‐BD2 binder with a K_D_ of 1 nm and over 2000‐fold selectivity against BRD4.^[^ ^19^ ^]^ The co‐crystal structure of TAF1‐BD2 in complex with GNE‐371 (PDB ID: 6DF7) revealed that the benzimidazole ring forms a hydrogen bond interaction with the gatekeeper Tyr1589 residue and the morpholine moiety of GNE‐371 is solvent‐exposed Figure 1b.^[^ ^19^ ^]^ We replaced the solvent‐exposed morpholine group with the piperazine group which serves as a handle for linker installation. We designed and synthesized a series of putative TAF1‐recruiting p53Y220C AceTACs (1–12) using either an alkylene linker or polyethylene glycol (PEG) linker (Figure 1c,d; Schemes S1 and S2, Supporting Information).

To evaluate these putative TAF1‐recruiting p53Y220C AceTACs, we utilized an NCI‐H1299 cell line which is endogenously p53‐null to stably express the FLAG tagged p53Y220C mutant, termed H1299‐p53Y220C. We observed that both PK9328 and GNE‐371 at 5 µm (8 h treatment) did not significantly induce acetylation of p53Y220C lysine 382 residue (p53K382ac) in H1299‐p53Y220C cell line (Figure 1e,f). On the other hand, compound 2 (MS172) at 5 µm (8 h treatment) induced the most significant increase in the p53K382ac level (≈10‐fold) among the 12 AceTAC compounds tested (Figure 1e,f). Notably, TAF1‐based p53Y220C AceTACs with a longer alkylene linker (such as 8‐carbon linker) or a PEG linker did not induce significant p53Y220C K382 acetylation (Figure 1e,f). Overall, we identified MS172 as the most effective TAF1‐recruiting p53Y220C AceTAC from this SAR study and selected this compound for further characterization.

### 2.2 MS172 Induces p53Y220C Acetylation in a Concentration‐ and Time‐Dependent Manner in Endogenously p53Y220C‐Expressing Cells

**Figure 2:**
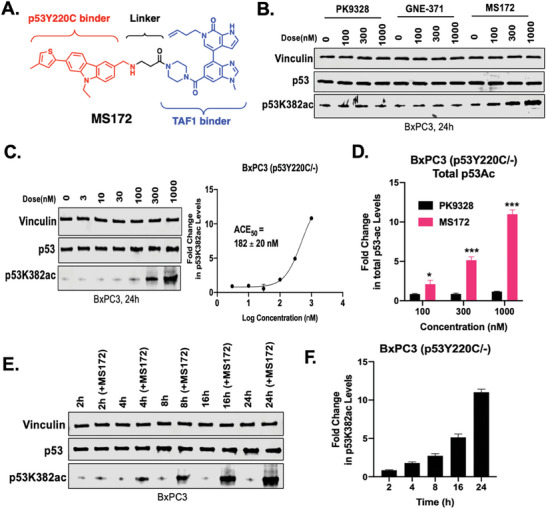
MS172 induces p53Y220C acetylation in a concentration‐ and time‐dependent manner in BxPC3 cells. A) Chemical structure of MS172. B) WB results of the p53K382ac level in BxPC3 cells treated with PK9328, GNE‐371, or MS172 at 0, 100, 300, or 1000 nm for 24 h. The results shown are representative of three independent experiments. Total cell lysate was used for WB and vinculin was used as a loading control. C) Left: WB results of the p53K382ac level in BxPC3 cells treated with MS172 at 0, 3, 10, 30, 100, 300 or 1000 nm for 24 h. The results shown are representative of three independent experiments. Total cell lysate was used for WB and vinculin was used as a loading control. Right: quantification of the fold change of the p53K382ac level shown on the left and its biological repeats. The results shown are the mean values ± SD from three independent experiments. D) Total p53 lysine acetylation in BxPC3 cells treated with PK9328 or MS172 at 100, 300, or 1000 nm for 24 h. A p53 mouse monoclonal antibody (DO‐1) was coated onto the microwells of an ELISA plate. After incubation with total cell lysates for 2 h, p53 was captured by the coated antibody. Following extensive washing, acetylated‐lysine rabbit monoclonal antibody was added to detect the total acetylated lysine on p53. An anti‐rabbit IgG with horse‐radish‐peroxidase (HRP) linked antibody was then used to recognize the bound detection antibody. Vehicle treatment was used to normalize the fold change of the total p53 lysine acetylation level. The results shown are the mean values ± SD from three independent experiments. ^*^ *p* <.05, ^***^ *p* <.001. E) WB results of the p53K382ac level in BxPC3 cells treated with 1000 nm of MS172 at the indicated time point. The results shown are representative of two independent experiments. Total cell lysate was used for WB and vinculin was used as a loading control. F) Quantification of the fold change of the p53K382ac level from the WB results shown in panel E and its biological repeat. Vehicle treatment from each specific time point was used to normalize the fold change of the p53K382ac level.

We next assessed the effect of MS172 on inducing p53Y220C K382 acetylation in the endogenously p53Y220C‐expressing BxPC3 (p53Y220C/‐) cell line. Compared to PK9328 and GNE‐371, MS172 induced significant p53Y220C K382 acetylation, even at 300 nm, in BxPC3 cells (**Figure** **2**a,b). We determined that the p53K382ac induced by MS172 in BxPC3 cells is concentration‐dependent with an ACE_50_ (the concentration at which 50% of p53Y220C is acetylated) of 182 ± 0 nm (with the acetylation level induced by MS172 at 1000 nm as the maximum response, Figure 2c). To assess whether MS172 induces total p53 acetylation, we employed an enzyme‐linked immunosorbent assay (ELISA) that detects endogenous levels of total acetylated p53. Compared to PK9328, MS172 significantly induced total p53 lysine acetylation, even at 100 nm (Figure 2d). In addition, MS172 induced p53K382 acetylation in a time‐dependent manner. It increased the p53K382ac level by about two‐fold at 4 h and by ≈10‐fold at 24 h (Figure 2e,f). Overall, these results demonstrate that MS172 effectively induces p53Y220C acetylation in a concentration‐ and time‐dependent manner in p53Y220C‐harboring cells.

### 2.3 MS172 Induces Ternary Complex Formation Between p53Y220C and TAF1

**Figure 3:**
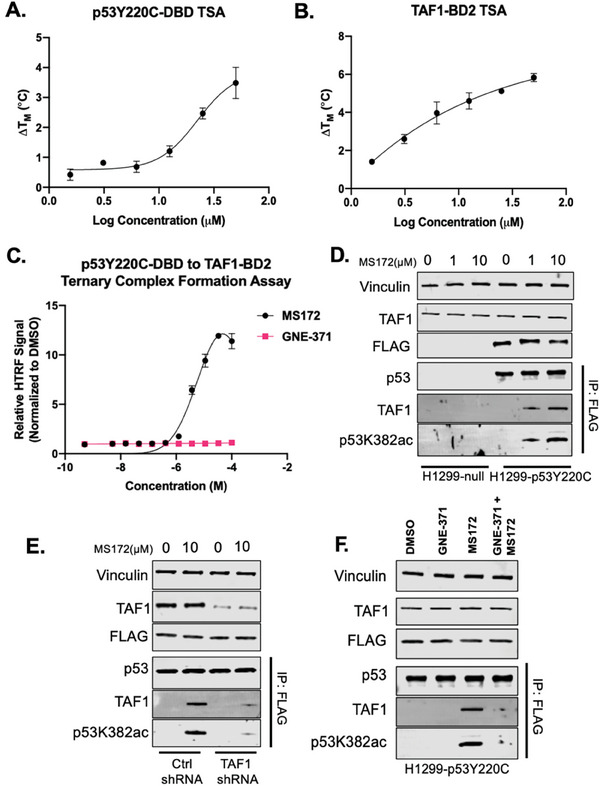
MS172 induces ternary complex formation between p53Y220C and TAF1. Thermal shift assay (TSA) results of p53Y220C‐DBD A) and TAF1‐BD2 B) induced by MS172. The results shown are the mean values ± SD from three biological repeats. C) FRET‐based biochemical assay results for GNE‐371‐ and MS172‐mediated ternary complex formation between GST‐TAF1‐BD2 and p53Y220C‐DBD‐His. Briefly, anti‐GST and anti‐His HTRF antibody pairs bind to GST‐tagged TAF1‐BD2 and His‐tagged p53Y220C‐DBD. A HTRF signal is generated only if the compound binds and the ternary complex is formed and a signal is normalized to the DMSO treatment. The results shown are the mean values ± SD from two biological repeats. D) Representative WB results of MS172‐mediated p53Y220C‐TAF1 interaction via p53‐FLAG pull‐down in H1299‐p53‐null and H1299‐p53Y220C cells treated with MS172 at 0, 1, or 10 µm for 24 h. The results shown are representative of two independent experiments. Total cell lysate was used for WB and vinculin was used as a loading control. E) Representative WB results of p53‐FLAG pull‐down after treatment of H1299‐p53Y220C cells with DMSO or MS172 at 10 µm for 24 h with either control shRNA or TAF1 shRNA. The results shown are representative of two independent experiments. Total cell lysate was used for WB and vinculin was used as a loading control. F) WB results of p53‐FLAG pulldown after treatment of H1299‐p53Y220C cells with GNE‐371 alone at 10 µm, MS172 alone at 3 µm, or GNE‐371 pretreatment at 10 µm for 4 h, followed by MS172 treatment at 3 µm for 20 h. The results shown are representative of two independent experiments. Total cell lysate was used for WB and vinculin was used as a loading control.

To determine whether MS172 induces a ternary complex formation between p53Y220C and TAF1, we first assessed the target engagement profile of MS172. We expressed and purified both p53Y220C‐DBD and TAF1‐BD2 and performed a protein thermal shift assay to determine the binding and stabilization effect of MS172 (**Figure** **3**a,b). We observed a concentration‐dependent thermal stability shift (ΔT_M_) of both proteins by MS172 with a ΔT_M_ of 3.5 and 5.8 °C for p53Y220C‐DBD and TAF1‐BD2, respectively, at 50 µm (Figure 3a,b). To further assess selectivity against other homologous bromodomain‐containing proteins, we also expressed and purified BRD4‐BD2, CBP‐BD, and PCAF‐BD (Figure S1, Supporting Information). Even at a higher concentration (100 µm), we found that MS172 did not stabilize bromodomains of BRD4, CBP, and PCAF (Figure S2, Supporting Information). In summary, MS172 stabilized p53Y220C and bound to TAF1 selectively over several other bromodomain‐containing proteins.

Next, we performed a Time‐resolved Fluorescence Resonance Energy Transfer (TR‐FRET) biochemical assay to assess the protein‐protein interaction between p53Y220C‐DBD and TAF1‐BD2 in the presence of MS172 (Figure 3c). We observed that MS172 induced ternary complex formation between p53Y220C‐DBD and TAF1‐BD2 while GNE‐371 did not (Figure 3c). We also performed immunoprecipitation (IP) experiments to pull down FLAG‐tagged p53Y220C to assess the ternary complex formation between p53Y220C and TAF1 intracellularly (Figure 3d). As expected, MS172 induced an interaction between p53Y220C and TAF1 and induced p53K382ac in a concentration‐dependent manner in H1299‐p53Y220C cells, whereas no interaction was observed in H1299‐p53‐null cells (Figure 3d). To further confirm that p53Y220C K382 acetylation depends on TAF1, we knocked down TAF1 using shRNA in the H1299‐p53Y220C cell line and monitored the p53Y220C‐TAF1 interaction and p53Y220C K382 acetylation induced by MS172 (Figure 3e). Upon treatment with MS172, there was an induction of the p53K382ac level in control shRNA‐treated cells concurrent with the interaction between p53Y220C and TAF1 (Figure 3e). On the other hand, the interaction between p53Y220C and TAF1 and p53Y220C K382 acetylation in the TAF1 shRNA‐treated cells was significantly diminished (Figure 3e), thereby confirming that the MS172‐mediated p53Y200C K382 acetylation is dependent on TAF1 interaction. Finally, we performed a competition rescue experiment to further validate the ternary complex formation between p53Y220C and TAF1 (Figure 3f). By pretreating H1299‐p53Y220C cells with 10 µm of GNE‐371 for 4 h, the p53Y220C‐TAF1 interaction was significantly abolished and the p53K382ac level induced by MS172 was significantly reduced Figure 3f. To further confirm that MS172 does not recruit p300, we performed a FLAG‐tag pulldown experiment with MS78 as a positive control. As expected, MS78 induced a ternary complex interaction between p53Y220C and p300 acetyltransferase, whereas MS172 did not induce any ternary complex formation between p53Y220C and p300 acetyltransferase (Figure S3, Supporting Information). Altogether, we confirmed that MS172‐induced p53Y220C K382 acetylation depends on the ternary complex formation between p53Y220C and TAF1 acetyltransferase by utilizing biochemical, FLAG‐IP, knockdown, and rescue experiments in isogenic cell lines.

### 2.4 MS172 Effectively Inhibits The Proliferation in p53Y220C‐Harboring Cancer Cells and is More Potent Than the p300/CBP‐Recruiting AceTAC MS78

**Figure 4:**
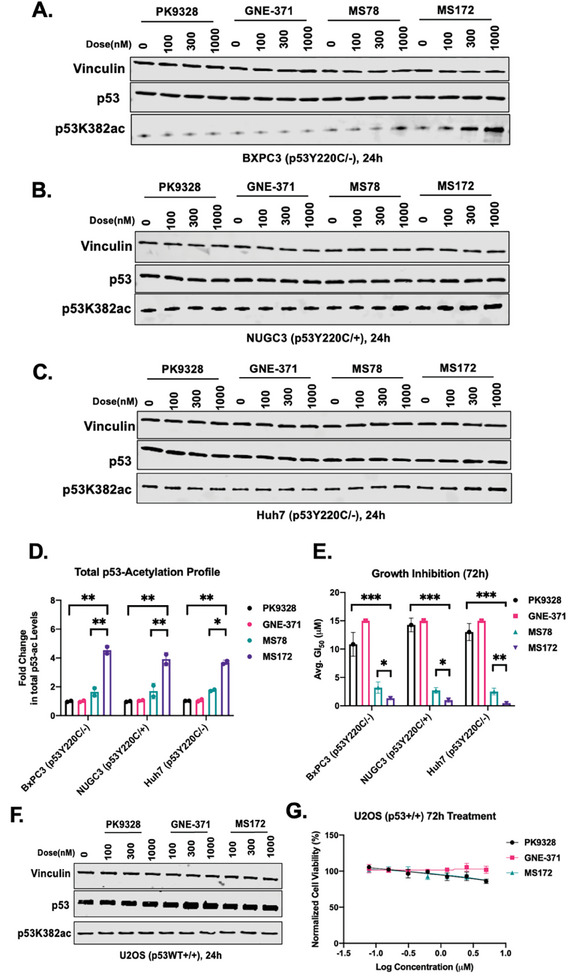
MS172 effectively acetylates p53Y220C and inhibits the growth in p53Y220C‐harboring cancer cell lines and is non‐toxic in p53 WT cells. WB results of PK9328, GNE‐371, MS78, and MS172 in A) BxPC3, B) NUGC3, and C) Huh7 cells treated with the indicated compound at 0, 100, 300, or 1000 nm for 24 h. The results shown are representative of two independent experiments. Total cell lysate was used for WB and vinculin was used as a loading control. D) Total p53 lysine acetylation in BxPC3, NUGC3 and Huh7 cells treated with PK9328, GNE‐371, MS78, or MS172 at 1000 nm for 24 h. Vehicle treatment was used to normalize the fold change of the total p53 lysine acetylation level. The results shown are the mean values ± SD from three independent experiments. ^*^ *p* <.05, ^**^ *p* <.01. E) Cell viability of PK9328, GNE‐371, MS78 and MS172 in BxPC3, NUGC3, and Huh7 cells. The cells were treated with DMSO or the indicated compound for 72 h. The mean values ± SD from three biological experiments (each in technical triplicates) are shown. GraphPad Prism 8 was used in the analysis of raw data. F) WB results of PK9328, GNE‐371, and MS172 in U2OS cells treated with the indicated compound at 0, 100, 300, or 1000 nm for 24 h. The results shown are representative of two independent experiments. Total cell lysate was used for WB and vinculin was used as a loading control. G) Cell viability of PK9328, GNE‐371, and MS172 in U2OS cells. The cells were treated with the indicated compound for 72 h. The mean value ± SD for each concentration (in technical triplicates from two biological experiments) is shown in the curves. GraphPad Prism 8 was used in the analysis of raw data.

Next, we compared the effects of MS172 to the p300/CBP‐recruiting AceTAC MS78 on inducing p53Y220C acetylation and their anti‐proliferative activity in multiple p53Y220C‐harboring cancer cell lines (BxPC3 (p53Y220C/‐), NUGC3 (p53Y220C/+), and Huh7 (p53Y220C/‐)). First, we observed that MS172 induced p53Y220C K382 acetylation more effectively than MS78 in BxPC3, NUGC3, and Huh7 cells (**Figure** **4**a–c). As expected, both PK9328 and GNE‐371 did not increase the p53K382ac level compared to the baseline at the concentrations tested (Figure 4a–c). We also assessed the total p53 lysine acetylation induced by MS172 and saw a significant upregulation compared to PK9328, GNE‐371, and MS78 (Figure 4d). To examine the correlation between p53Y220C acetylation and anti‐proliferative activity, we performed cell viability experiments for MS172, MS78, PK9328, and GNE‐371 in all three cell lines (Figure 4e). As expected, MS172 displayed the most potent anti‐proliferative activity and is more potent than MS78 in all three p53Y220C‐harboring cell lines (Figure 4f; Table S1 Supporting Information). Altogether, MS172 is more effective than MS78 in both inducing p53Y220C acetylation and suppressing the proliferation in multiple p53Y220C‐harboring cancer cell lines.

We then tested the cytotoxicity profile of MS172 in both p53 wildtype (WT) cell lines and normal cells. MS172 did not induce any significant p53 K382 acetylation at the concentrations tested in U2OS, a p53 WT cell line, similar to PK9328 and GNE‐371 (Figure 4f). Additionally, MS172 as well as PK9328 and GNE‐37 did not display a significant cell growth inhibition effect in U2OS cells (Figure 4g). We also assessed the effect MS172 on the growth of normal prostate PNT2 cells and did not observe significant growth inhibition (Figure S4, Supporting Information). Finally, we evaluated the in vivo mouse pharmacokinetic (PK) properties of MS172 (Figure S5, Supporting Information). We determined the plasma concentrations of MS172 in Swiss albino mice following a single intraperitoneal (IP) administration of 50 mg kg^−1^. The plasma concentrations of MS172 were ≈ 0.7 µm for the first 2 h and maintained above 0.5 µm for 8 h post the IP injection (Figure S5, Supporting Information). We did not observe significant toxicity of MS172 in vivo and the compound was well tolerated in the treated mice. Future studies to assess in vivo efficacy of MS172 are warranted. Taken together, these results indicate that MS172 is non‐toxic in p53 WT cancer cells and normal cells and has sufficient mouse PK properties to be used as a chemical biology tool to investigate the role of p53Y220C acetylation in vivo.

### 2.5 Novel Regulation of Metallothionine Proteins by MS172 in BxPC3 Cells

**Figure 5:**
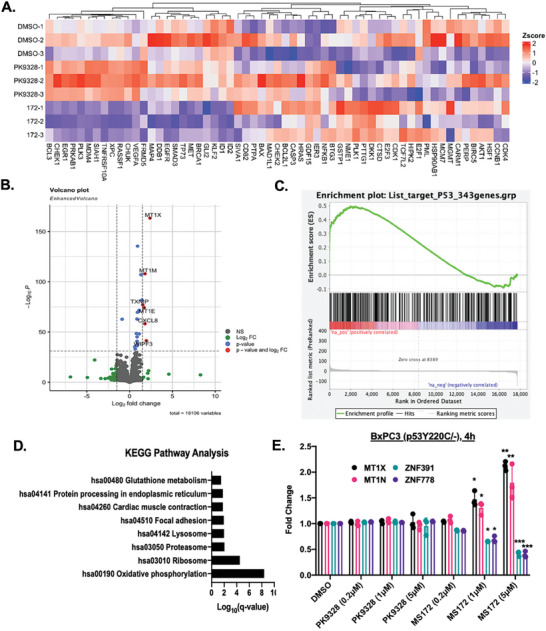
MS172‐mediated p53Y220C acetylation induces regulation of metallothionine proteins in BxPC3 cells. A) Heatmap enrichment in high confidence p53‐target genes. BxPC3 cells were treated with DMSO, PK9328 or MS172 at 5 µm for 4 h in triplicates. B) Volcano plot of differential gene expression (DGE) of upregulated proteins and pathways upon MS172 treatment compared to PK9328 treatment after normalization with DMSO treatment. C) Enrichment plot of significant genes upregulated from the 343 high‐confidence p53‐target genes (q‐value <.01, normalized enrichment score (NES) = 1.49) in BxPC3 cells treated with 5 µm of MS172 compared to 5 µm of PK9328 treatment for 4 h in triplicates. D) KEGG pathway analysis of upregulated pathways upon 5 µm of MS172 treatment compared to 5 µm of PK9328 for 4 h in triplicates. E) RT‐qPCR of *MT1X*, *MT1N*, *ZNF391*, and *ZNF778* expression in BxPC3 cells treated with DMSO, PK9328, or MS172 at 0.2, 1, or 5 µm for 4 h from three independent experiments. The mRNA expression for each gene was first normalized to internal GAPDH and then calculated relative to the DMSO control. ^*^ *p* <.05, ^**^ *p* <.01, ^***^ *p* <.001.

Finally, we evaluated the downstream signaling difference between MS172 and the parent p53Y220C stabilizer PK9328. While we previously assessed the effect of MS78 in H1299‐p53Y220C cells,^[^ ^5^ ^]^ we did not evaluate 1) the effect of p53Y220C AceTAC in a cell line that endogenously expresses p53Y220C, and 2) which genes are regulated first after p53Y220C acetylation. Thus, we performed an RNA‐sequencing (RNA‐seq) study in BxPC3 cells treated with DMSO, PK9328, or MS172 at 5 µm for 4 h, a relatively early time point (**Figure** **5**). Interestingly, while PK9328 induced the upregulation of typical p53‐regulated target genes such as *BAX*, *CHEK1*, and *CDKN1A*, it also induced the expression of *NFKB1*, *KRAS*, and *MDM2* genes at this early time point (Figure 5a), which is consistent with previous RNA‐seq and RT‐qPCR results of p53Y220C stabilizers.^[^ ^20^, ^21^, ^22^ ^]^ On the other hand, at this early time point, MS172 treatment significantly upregulated *MT1X, MT1N, PLK1*, and *DKK1* genes (Figure 5a). Particularly, we compared the gene expression profile of MS172 to PK9328 (after normalization with DMSO‐treated samples) and observed a significant upregulation of these metallothionine proteins while a downregulation of zinc‐finger containing proteins (log_2_fold change >2, *p*‐value <0.05, Figure 5b). It was reported previously that WT p53 can bind metallothionine proteins and inactivation of WT p53 leads to a downregulation of metal response elements (MREs).^[^ ^23^, ^24^ ^]^ However, to the best of our knowledge, the regulation of metallothionine proteins by p53Y220C has not been previously reported. Furthermore, unbiased analysis using differential gene expression (DGE) and enrichment in KEGG pathways revealed that MS172 treatment led to the upregulation of proteins involved in oxidative phosphorylation compared to PK9328 (q‐value = 0.005, normalized enrichment score (NES) = 1.49) (Figure 5c,d). These results are consistent with previous studies which showed that metallothionine proteins are involved in oxidative stress and inhibition of metallothionine proteins can increase cancer cell growth by regulating zinc‐finger proteins.^[^ ^25^, ^26^ ^]^ To validate the RNA‐seq results, we performed a subsequent RT‐qPCR study in BxPC3 cells treated with DMSO, PK9328, or MS172 at different concentrations (Figure 5e). We observed a concentration‐dependent upregulation of *MT1X* and *MT1N* genes and a simultaneous downregulation of *ZNF391* and *ZNF778* genes at 4 h time point induced by MS172, while PK9328 treatment led to no changes compared to the DMSO control. We also determined whether these novel pathways are regulated in other p53Y220C‐harboring cancer cell lines as well as p53‐WT and p53‐null cell lines (Figure S6, Supporting Information). MS172 indeed induced the expression of *MT1X* and *MT1N* genes while suppressing *ZNF391* and *ZNF778* genes at 4 h time point in NUGC3 cell line, but not in p53‐WT (HCT116) and p53‐null (NCI‐H1299) cell lines (Figure S6, Supporting Information). It should be noted while these short‐treatment RNA‐seq and RT‐qPCR studies revealed a novel mechanism of action for MS172, additional studies are warranted to assess how the changes in these gene pathways at an early time point lead to differential gene expression at later time points compared to the parent p53Y220C stabilizer. Moreover, further studies to determine whether p53Y220C acetylation by AceTAC leads to novel DNA‐binding by p53Y220C are also warranted. In summary, we observed a novel regulation of metallothionine proteins by MS172‐induced p53Y220C acetylation compared to the parent p53Y220C stabilizer.

## 3 Conclusion

In this study, we discovered and characterized the first‐in‐class TAF1‐recruiting p53Y220C AceTAC, MS172. MS172 effectively induced p53Y220C K382 acetylation and increased total p53Y2220C acetylation in a concentration‐ and time‐dependent manner. We validated that the p53Y220C K382 acetylation induced by MS172 depends on the ternary complex formation between p53Y220C and TAF1 using a battery of biochemical, immunoprecipitation, knockdown, and rescue experiments. We also determined that MS172 effectively induced p53Y220C K382 acetylation and inhibited the proliferation in multiple p53Y220C‐harboring cancer cell lines. Notably, MS172 is more potent than the parent p53Y220C binder PK9328, the TAF1 bromodomain binder GNE‐371, and our previously reported p300/CBP‐recruiting p53Y220C AceTAC MS78 in inducing p53Y220C K382 acetylation and total p53 acetylation in multiple p53Y220C‐harboring cancer cell lines. Importantly, our TAF1‐recruiting p53Y220C AceTAC MS172 is effective in suppressing the tumor cell growth in multiple cancer cell lines including pancreatic (BxPC3), gastric (NUGC3), and liver (Huh7), and is more potent than PK9328, GNE‐371, and MS78, providing a potential therapeutic approach for the treatment of p53Y220C‐based solid cancers. Furthermore, MS172 is non‐toxic in p53 WT and normal cells and is bioavailable in mice via IP administration, thus making it suitable for in vivo efficacy studies. Lastly, we uncovered novel upregulation of metallothionein proteins mediated by MS172‐induced p53Y220C acetylation using RNA‐seq and RT‐qPCR studies.

While our proof‐of‐concept study shows potential therapeutic utilities of TAF1‐recruiting p53Y220C AceTAC, it should be noted that the lead compound from this proof‐of‐concept study, MS172, which displayed superior antiproliferative effects than the parent p53Y200C stabilizer, is not a drug candidate. Further optimization of MS172, which is a useful tool compound, is needed to develop a clinical candidate for translating this therapeutic approach in the clinic. It is likely that p53Y220C AceTAC will provide a complementary therapeutic approach to Rezatapopt (PC14586), a p53Y220C small‐molecule stabilizer in clinical development, which has shown some efficacy in p53Y220C‐ harboring solid cancers.^[^ ^27^ ^]^ Currently, AceTAC targeting p53 is limited to p53Y220C, due to the lack of bona fide small‐molecule binders for WT p53 or other p53 mutants. However, in the future, upon the development of bona fide small‐molecule binders for WT p53 or other p53 mutants by the scientific community, novel AceTACs targeting WT p53 or other p53 mutants will be developed, which could augment p53‐acetylation mediated tumor suppression for the treatment of cancer.

Overall, we have demonstrated that the TAF1 acetyltransferase can be harnessed for AceTAC development, thus expanding the very limited repertoire of the KATs that can be hijacked for inducing targeted protein acetylation. We also provide MS172, a valuable chemical biology tool and a potential therapeutic, to the scientific community to further investigate the role of p53Y220C acetylation in cancer.

## Conflict of Interest

The authors declare the following competing financial interest(s): J.J. is a cofounder and equity shareholder in Cullgen, Inc., a scientific cofounder and scientific advisory board member of Onsero Therapeutics, Inc., and a consultant for Cullgen, Inc., EpiCypher, Inc., Accent Therapeutics, Inc, and Tavotek Biotherapeutics, Inc. The Jin laboratory received research funds from Celgene Corporation, Levo Therapeutics, Inc., Cullgen, Inc. and Cullinan Oncology, Inc.

## Author Contributions

M.K. and X.H. contributed equally to this work.

## Supporting information



Supporting Information

## Data Availability

The data that support the findings of this study are available from the corresponding author upon reasonable request.
