# Supplementary material for: Harnessing the TAF1 Acetyltransferase for Targeted Acetylation of the Tumor Suppressor p53
Source: Adv Sci (Weinh). 2024 Dec 24;12(7):2413377. doi: 10.1002/advs.202413377 (PMC11831463; doi:10.1002/advs.202413377)
Supplement: Supplementary file 1 — Supporting Information [file ADVS-12-2413377-s001.docx]

Supporting Information

Harnessing the TAF1 Acetyltransferase for Targeted Acetylation of the Tumor Suppressor p53

Md Kabir^1,2,3#^, Xiaoping Hu^1,2,3#^, Tiphaine C. Martin^3^, Dmitry Pokushalov^1,2,3^, Yong Joon Kim^2^, Yiyang Chen^3^, Yue Zhong^1,2,3^, Qiong Wu^1,2,3^, Jerry E. Chipuk^3^, Yi Shi^2^, Yan Xiong^1,2,3^, Wei Gu^4^, Ramon E. Parsons^3^, and Jian Jin^1,2,3*^

^1^Mount Sinai Center for Therapeutics Discovery, Icahn School of Medicine at Mount Sinai, New York, NY 10029, USA

^2^Department of Pharmacological Sciences, Icahn School of Medicine at Mount Sinai, New York, NY 10029, USA

^3^Department of Oncological Sciences, Tisch Cancer Institute, Icahn School of Medicine at Mount Sinai, New York, NY 10029, USA

^4^Institute for Cancer Genetics, and Department of Pathology and Cell Biology, and Herbert Irving Comprehensive Cancer Center, Vagelos College of Physicians & Surgeons, Columbia University, New York, NY, 10032, USA

^#^ These authors contributed equally to this work

* Correspondence: Jian Jin: [jian.jin@mssm.edu](mailto:jian.jin@mssm.edu)

27 pages

Supporting Information Figures S1-S6

Supporting Information Table S1

Materials and Methods

Chemical Synthesis


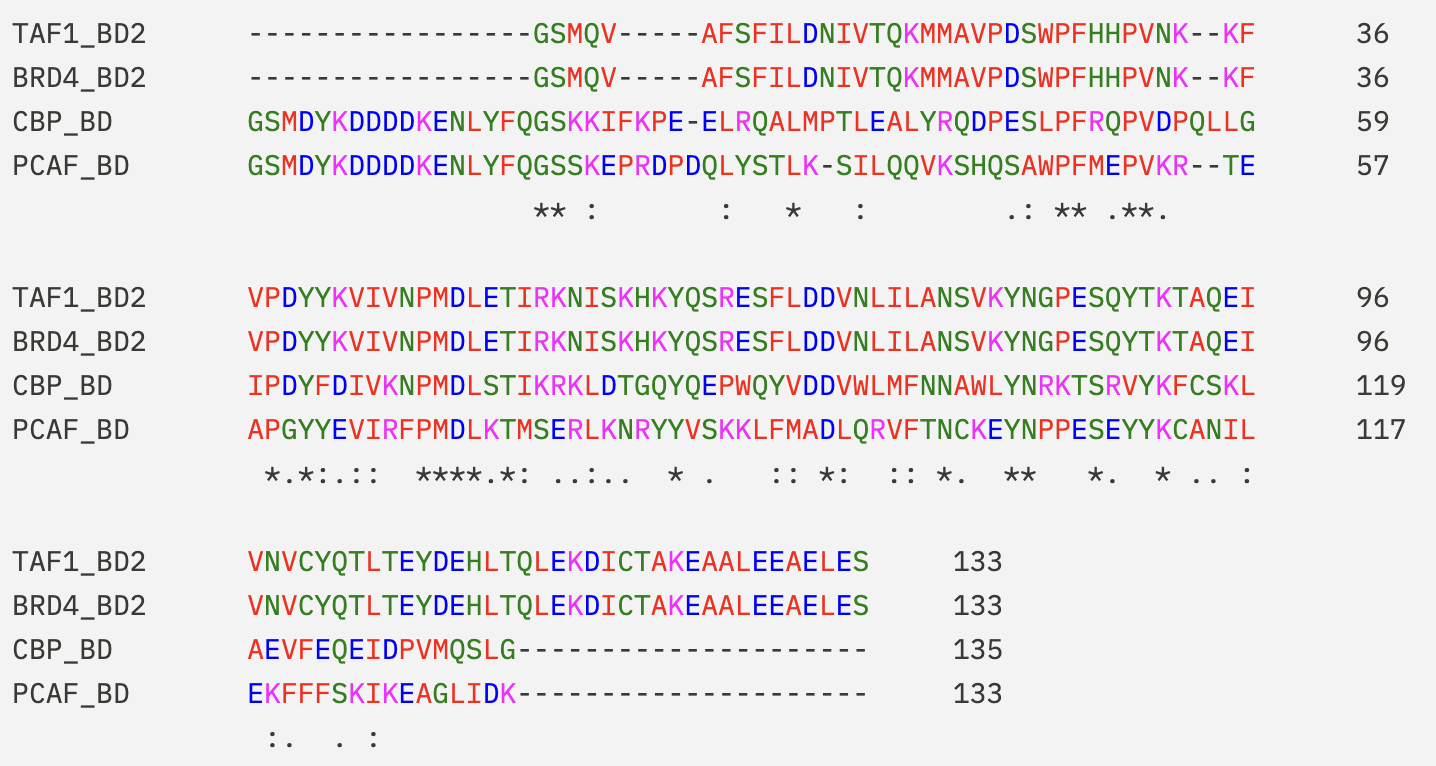


**Supporting Information Figure S1**. **Sequence comparison of TAF1 and other bromodomain-containing proteins.** The sequence of TAF1-BD2 is compared to BRD4-BD2, CBP-BD and PCAF-BD. (*) denotes positions with fully conserved residues, (:) denotes conservation of residues with strongly similar properties, and (.) denotes conservation of residues with weakly similar properties.

**Supporting Information Figure S2**. **TSA results of MS172 at 100 μM against other bromodomain-containing proteins.** Results shown are the mean values from two independent experiments.


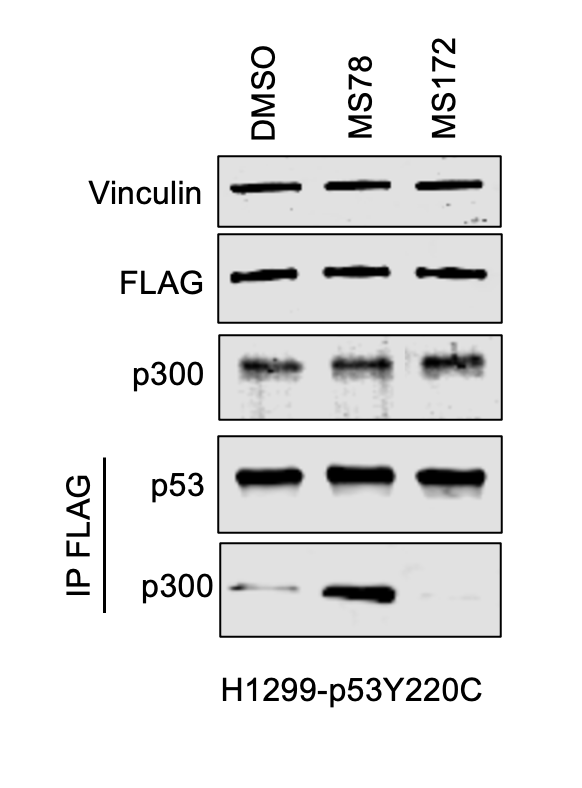


**Supporting Information Figure S3**. **MS172 does not induce an interaction between p53Y220C and p300 acetyltransferase.** Representative WB results of p53-FLAG pull-down after treatment of H1299-p53Y220C cells with DMSO, MS78 or MS172 at 10 μM for 24 h. Total cell lysate was used for WB and vinculin was used as a loading control. The results shown are representative of two independent experiments.

**Supporting Information Figure S4**. **MS172 does not significantly inhibit the growth in PNT2 normal prostate cells.** PNT2 cells were treated with the indicated compound at the indicated concentration for 72 h. The mean value ± SD for each concentration point (in technical triplicates from three biological experiments) is shown. GraphPad Prism 8 was used in analysis of raw data.


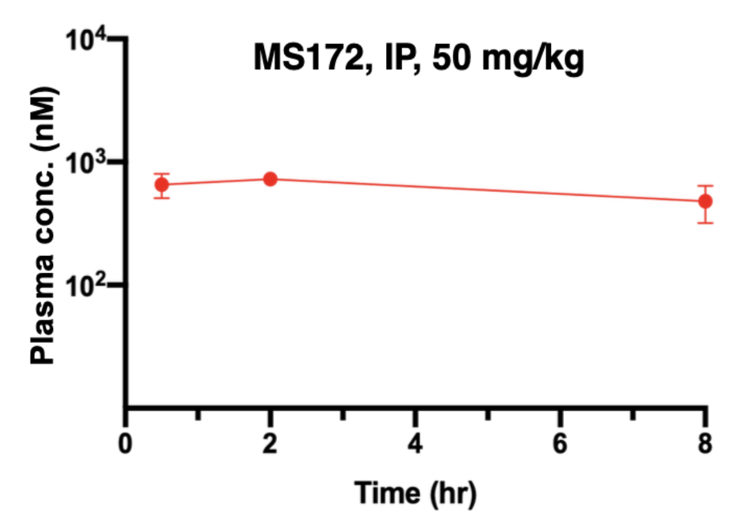


**Supporting Information Figure S5.** **MS172 is bioavailable in an *in vivo* mouse pharmacokinetic study.** Plasma concentrations of MS172 over 8 h in Swiss albino mice were determined following a single 50 mg/kg intraperitoneal (IP) injection. Experiments were performed in biological triplicates. Each point represents the mean concentration ± SEM.

**Supporting Information Figure S6. Novel mechanism of action of MS172.** RT-qPCR results of *MT1X, MT1N, ZNF391*, and *ZNF778* expression in NUGC3, HCT116 and NCI-H1299 cells treated with DMSO, PK9328 or MS172 at 5 μM for 4 h from two independent experiments. The mRNA expression for each gene was first normalized to internal GAPDH and then calculated relative to the DMSO control.

**Supporting Information Table S1: GI_50_ values of MS172, MS78, PK9328 and GNE-371 in BxPC3, NUGC3 and Huh7 cells.**


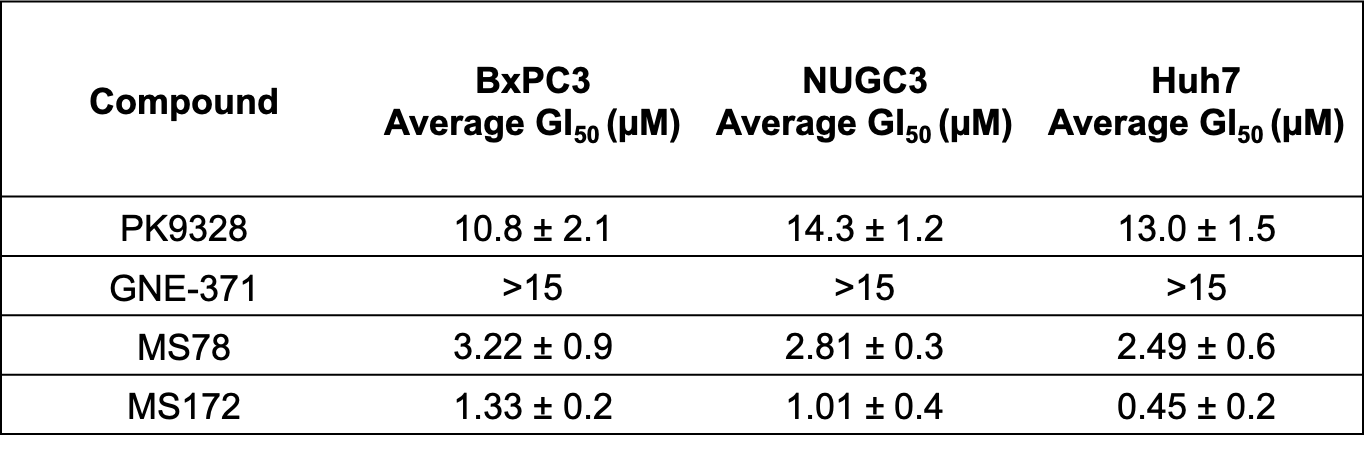


Cell viability results of MS172, MS78, PK9328, and GNE-371 in BxPC3, NUGC3 and Huh7 cells. The cells were treated with the indicated compound for 72 h. The mean GI_50_ values ± SD from three biological experiments (in technical triplicates) are shown.

**Materials & Methods**

Cell Lines, Tissue Culture and Transfection.

NCI-H1299 (CRL-5803), BxPC3 (CRL-1687) and U2OS (HTB-96) were purchased from the American Tissue Culture Collection (ATCC, Manassas, VA). In addition, NUGC-3 (JCRB0822) and Huh7 (JCRB0403) were obtained from the Japan Health Science Research Resources Bank (JCRB, Japan). Normal prostate epithelium PNT2 cell line (95012613) was purchased from Sigma-Aldrich (Burlington, Massachusetts). NCI-H1299, BxPC3, NUGC-3 and PNT2 cells were cultured in RPMI 1640 medium (Thermo Fisher Scientific Inc., Waltham, Massachusetts) supplemented with 10% heat-inactivated fetal bovine serum Gibco^TM^ (FBS) (Life Technologies, Grand Island, NY) and 1% Gibco^TM^ Penicillin/Streptomycin (Life Technologies, Grand Island, NY). Huh7 cells were cultured in DMEM Medium (Thermo Fisher Scientific Inc., Waltham, Massachusetts) supplemented with 10% heat-inactivated FBS and 1% Penicillin/Streptomycin. U2OS cells were cultured in McCoy's 5A (Modified) Medium (Thermo Fisher Scientific Inc., Waltham, Massachusetts) supplemented with 10% heat-inactivated FBS and 1% Penicillin/Streptomycin. All cells were incubated at 37 °C in a standard humidified incubator containing 5% CO_2_ and 95% O_2_.

For shRNA transfection, control and TAF1 shRNA (sc-106597-SH) were purchased from Santa Cruz Biotechnology. Briefly, H1299-p53Y220C cells were seeded on 6-well plates at a density of 3 × 10^5^ cells/well (~75–80% confluency). Cells were transfected the following day with Lipofectamine 3000 transfection reagent (L3000008, ThermoFisher Scientific) and harvested 48 h after transfection. For the construction of the FLAG-p53Y220C stable cell line, NCI-H1299 cells were transfected with sequence verified pcDNA3.1(+)-N-DYK vectors containing specific ORFs (Genscript, Piscataway, NJ) and selected with G418 for 14 days.

Antibodies and Immunoblotting.

Total cell lysate was used for western blots, as previously described.^1^ The following primary antibodies were used in the study: Vinculin (Cell Signaling Technology [CST], 13901), p53 (CST, 2527), acetyl-p53 (lys-382) (CST, 2525), acetylated-lysine antibody (CST, 9441), TAF1 (D6J8B) (CST, 12781), p53 polyclonal antibody (Proteintech, 10442-1-AP). Blots were imaged using fluorescence-labeled secondary antibodies on LI-COR Odyssey CLx Imaging Systems.

Protein expression and purification.

A stabilized version of the human p53 DBD (residues 94–312; M133L/V203A/N239Y/N268D) with an additional Y220C mutation was expressed and puriﬁed as previously described.^2^ TAF1-BD2, BRD4-BD2, CBP-BD and PCAF-BD were expressed and puriﬁed as previously described.^3^

Total p53 Acetylation ELISA Assay

PathScan® Acetylated p53 Sandwich ELISA Kit (Cat# 7236) was purchased from CST and used according to manufacturer’s instructions. Briefly, cell lysate after compound treatment was used. Absorbance signals were obtained with Infinite F PLEX plate reader (TECAN, Morrisville, NC) at 450 nm with 550 nm as reference wavelength after adding stop solution. GraphPad Prism 8 was used in the analysis of the total p53 lysine acetylation from three independent experiments.

Thermal Shift Assay.

Thermal shift assay was performed in 100-μl 96-well PCR plates. First 10× SYPRO® Orange dye (S6650, ThermoFisher Scientific) was prepared in 1xHEPES buffer. PK9328, GNE-371 or MS172 was diluted to the target concentration (50 μM) and titrated in a 2-fold serial dilution in the buffer. In 96-well PCR plates, 20 μL of 2.5 μM protein, 20 μL of drug titrant, and 10× SYPRO® Orange were combined and centrifuged to bring solutions to the bottom of the well and remove bubbles. The plates were then sealed with optically clear adhesive sheet and subjected to Applied Biosystems ViiA7 real-time PCR instrument for spectrometry. Fluorescence counts versus temperature for each experimental condition were plotted, and the melting temperature was determined by the greatest first derivative of the plot.

FLAG-immunoprecipitation.

Endogenous co-IP experiments were performed as described previously.^1^ Briefly, 2 ×10^6^ cells were seeded in a 10-cm dish, and treated with 0, 1 or 10 μM of MS172 for 24 h. Cells were then washed with ice-cold PBS twice and centrifuged at 200 xg for 3 min to get cell pellets which were then lysed with 300 μL of lysis buffer (20 mM HEPES, pH 7.4, 150 mM NaCl, 2 mM EDTA, 1% Triton X-100, and EDTA-free phosphotase and protease inhibitor) for 30 min on ice, and centrifuged at 14,000g for 10 min to get supernatant as cell lysate. The ANTI-FLAG® M2 affinity gel (Product #A2220, Sigma-Aldrich, Inc.) was used for the purification of FLAG-p53Y220C, according to the manufacturer’s guidelines. Following incubation of M2 affinity gel, protein was eluted using 3X FLAG® Peptide (Product #F4799, Sigma-Aldrich, Inc.) and supernatant was resuspended in 30 μL of 1 × SDS-PAGE Laemmli buffer for boiling. The protein samples were then tested by WB.

Cell Viability Assay.

The cell viability assay was performed as previously described.^1^ Briefly, 5x10^4^ cells were seeded per well into 96-well microplates. After 24 h, cells were treated with 3-fold serially diluted compounds in triplicate for 72 h. Cell viability was evaluated using WST-8 reagent (CK04, Dojindo). Absorbance signals were obtained with Infinite F PLEX plate reader (TECAN, Morrisville, NC) at 450 nm with 690 nm as reference wavelength after 3 h incubation at 37 °C. GraphPad Prism 8 was used in the analysis of GI_50_ values from the data of three independent experiments.

TR-FRET Assay for p53Y220C-DBD and TAF1-BD2 Protein-Protein Interaction.

The TR-FRET assay for determining ternary complex formation was performed by Reaction Biology Corp. (Malvern, PA). Briefly, 10 nM of TAF1-BD2 (GST-tagged) and 10 nM of p53Y220C-DBD (His-tagged) were prepared in 50 mM Tris pH 7.5, 100 mM NaCl, 0.05% CHAPS and 1 mM DTT buffer. GNE-371 or MS172 at 1% final DMSO concentration was incubated with the proteins at room temperature for 30 mins. 6.7 nM HTRF MAb Anti-6HIS Tb cryptate Gold and 0.5 nM HTRF MAb Anti GST-d2 were used as the detection antibody.

Quantitative Reverse Transcriptase Polymerase Chain Reaction (RT-qPCR).

RT-qPCR was performed as previously described.^1^ Briefly, cells were treated with DMSO or indicated compounds at specific time points. Total RNA was extracted using the Monarch Total RNA Miniprep Kit (T2010S, New England Biolabs), and cDNA was generated using the SuperScript IV First-Strand Synthesis System (18091050, Thermo Fisher). qPCR was performed using the PowerUp SYBR Green Master Mix (Thermo Fisher Scientific, A25742) on Agilent Technologies Stratagene Mx3005p qPCR system. RxnReady™ premixed primer pairs for *MT1X, MT1N, PLK1* and *DKK1* were purchased from Integrated DNA Technologies, Inc. (Coralville, Iowa). *GAPDH* forward: 5′-ACAACTTTGGTATCGTGGAAGG-3′, *GAPDH* reverse: 5′-GCCATCACGCCACAGTTTC-3′ was used as controls. The mRNA expression for each target gene was first normalized to internal GAPDH and then calculated relative to the DMSO control. Experiments were performed in triplicates.

RNA-seq study.

The RNA-seq study was conducted as previously described.^1^ BxPC3 cells were treated with DMSO or 5 μM of MS172 or PK9328 for 4 h in triplicates. Cells were washed three times using ice-cold 1XPBS and subsequently pelleted by centrifuging at 12,000 rpm at 4°C. The pellet was flash frozen and sent to Azenta Life Sciences for further studies. The total RNA in 9 samples were extracted using Qiagen RNeasy Plus Mini Kit according to the protocols in the RNeasy Plus Mini Handbook published by Qiagen. RNA-seq libraries were constructed from the PolyA selected mRNA using the TruSeq RNA sample preparation guide (Illumina) and paired-end 150 base pairs sequencing on a HiSeq2500 system (Illumina) was performed at Azenta Life Sciences. The raw data in FASTQ format was analyzed as previously described.^1^

After quality control of FASTQ files using the FASTQC tool (version 0.11.7) (<http://www.bioinformatics.babraham.ac.uk/projects/fastqc/>), we trimmed low-quality bases (Phred < 10) and adapter sequences and then discarded short reads (length < 60 nt) using the bbduk tool (version 37.53) (<https://jgi.doe.gov/data-and-tools/bbtools/bb-tools-user-guide/>). When either forward or reverse of a paired-end read were discarded, we discarded the complete paired-end read. We quantified the expression of transcripts using cleaned paired-end reads with the Salmon tool (version 0.9.1)^4^ using the human reference transcriptome from The Cancer Genome Atlas (TCGA; GDC.h38 GENCODE v22).^5^ We performed the differential expression between DMSO, PK9328 or MS172 treated BxPC3 cells using the R tximport library (version 1.26.1) on R (version 4.2.2).^6^ We pre-filtered genes to keep only genes that had at least 10 reads in total (Ngene = 22,454) and then performed differential gene expression using the R DESeq2 library (version 1.38.3). We identified as differential expressed between two conditions when the *P* value adjusted was at 5% and log_2_(fold change) was more than 1. The heatmap was drawn using the R ComplexHeatmap library (version 2.14.0). We next performed GSEA to capture pathways perturbed towards both directions simultaneously using the 22,454 ranked genes identified in our dataset and annotated in ENSEMBL (version 94) against the 343 p53 target genes listed in previously reported study^7^ using GSEAPreranked (version 4.3.2.; number of permutations = 1000, no collapse) and the KEGG pathways using using R GAGE (version 2.48.0).

Data Availability.

RNA-seq data have been deposited in the GEO database (GEO accession no. GSE277017). There are no restrictions on data availability.

Mouse pharmacokinetic study.

Three male Swiss Albino mice were administered intraperitoneally with solution formulation of MS172 at a 50 mg/kg dose. The following formulation vehicle was used in the study: 10% v/v NMP, 10% v/v Solutol and 80% v/v normal Saline. Blood samples (approximately 60 μL) were collected from the three test mice at 0.5, 2 and 8 h. Plasma was harvested by centrifugation of blood and stored at -70 ± 10 ^o^C until analysis. Plasma samples were quantified by fit-for-purpose LC-MS/MS method. Compound concentrations in plasma at each time point are average values from 3 test mice. Error bars represent ± SEM. Experiments involving mice were performed according to the Institutional Animal Care and Use Committee (IACUC)-approved protocol.

Statistics and Reproducibility.

Experimental data are presented as the mean ± SD or SEM of three independent experiments unless otherwise noted. Statistical analysis was performed using an unpaired two-sided Student’s t test for comparing two sets of data with assumed normal distribution. The results for immunoblotting are representative of at least three biologically independent experiments unless otherwise noted. All statistical analyses and visualizations were performed using GraphPad (Prism v8.4.2) and BioRender.

Safety Statement

No unexpected or unusually high safety hazards were encountered during the synthesis of testing of the molecules described in the manuscript or SI.

**CHEMISTRY EXPERIMENTAL SECTION**

**Chemistry General Procedures.** All commercially available chemical reagents were used directly in syntheses without further purification. A Teledyne ISCO Combi*Flash* Rf^+^ instrument and HP C18 Redi*Sep* Rf reverse phase columns equipped with UV detector were used to conduct flash chromatography. All final compounds for biological evaluation were purified with preparative high-performance liquid chromatography (HPLC) on an Agilent Prep 1200 series with the UV detector set to 254 or 220 nm with a flow rate of 40 mL/min at room temperature. Crude samples were injected into a Phenomenex Luna 750 x 30 mm, 5 µ C18 column, with the gradient program set to 10% of methanol or acetonitrile (B) in H_2_O containing 0.1% TFA (A) progressing from 10 % to 100% of methanol or acetonitrile (B). The purity of all compounds for biological testing is > 95% assessed by HPLC-HRMS. All HPLC spectra was obtained by using an Agilent 1200 series system with DAD detector and a 2.1 mm x 150 mm Zorbax 300SB-C18 5 µ column for chromatography. Samples (0.5 µL) were injected onto a C18 column at room temperature with the flow rate of 0.4 mL/min. Chromatography was performed with the solvent as follows: water containing 0.1% formic acid was designated as Solvent A while acetonitrile containing 0.1% formic acid was designated as solvent B. The linear gradient was set such that 1% B was used from 0-1 min, 1-99% B from 1-4 min, and 99% B from 4-8 min. High-resolution mass spectra (HRMS) data was acquired in positive ion mode using an Agilent G1969A API-TOF with an electrospray ionization (ESI) source. All compounds were also characterized using either a Bruker (Billerica, MA) DRX Nuclear Magnetic Resonance (NMR) spectrometer (400 MHz, ^1^H NMR, 101 MHz ^13^C NMR). Chemical shifts for all compounds are reported in units of parts per million (ppm, δ) relative to residual solvent peaks. ^1^H NMR data are reported in the following format: chemical shift, multiplicity (s = singlet, d = doublet, t = triplet, q = quartet, m = multiplet), coupling constant, and integration. **Linkers 1 – 12^[1]^,** intermediate **I–2^[2]^** and intermediate **I–3^[2]^** were prepared following the reported procedures.

**Scheme S1**. **General synthetic route for preparing intermediate I-5***^a^*

*^a^*Reaction and conditions: (a) 4-Bromobut-1-ene, NaH, DMF, 0 ^o^C to rt, 2 h; (b) **I – 2**, Pd(dppf)Cl_2_, Cs_2_CO_3_, dioxane/H_2_O , 100 ^o^C, 3 h; (c) K_2_CO_3_, CH_3_I, DMF, 3 h; (d) NaOH, MeOH/H2O, 80 ^o^C, 2 h; (e) *tert*-butyl piperazine-1-carboxylate, HATU, DIPEA, DMF, rt; (f) TFA/DCM, rt.

**4-Bromo-6-(but-3-en-1-yl)-1-tosyl-1,6-dihydro-7*H*-pyrrolo[2,3-*c*]pyridin-7-one (I–1**). To a solution of 4-bromo-1-tosyl-1,6-dihydro-7*H*-pyrrolo[2,3-*c*]pyridin-7-one (950 mg, 2.57 mmol, 1.0 equiv) in DMF (6 mL) were added sodium hydride (123 mg, 3.08 mmol, 1.2 equiv) under 0 ^o^C ice bath for 15 min, then 4-bromobut-1-ene (555 µL, 5.14 mmol, 2 equiv) and the reaction solution was stirred at rt for 2 h. The resulting system was quenched with H_2_O and extracted with EA (50 mL* 3). The combined organic layers were concentrated in vacuo and the residue was purified by flash chromatography (hexane/ethyl acetate = 20%) to afford the desired intermediate **I-1** as white solid (400 mg, 37% yield). ^1^H NMR (400 MHz, Methanol-*d*_4_) δ 8.04 (d, *J* = 3.5 Hz, 1H), 7.90 (d, *J* = 8.0 Hz, 2H), 7.52 (d, *J* = 3.1 Hz, 1H), 7.36 (d, *J* = 8.0 Hz, 2H), 6.59 (d, *J* = 3.5 Hz, 1H), 5.82 – 5.50 (m, 1H), 4.96 – 4.89 (m, 2H), 4.04 – 3.83 (m, 2H), 2.46 – 2.30 (m, 5H).

**4-(6-(But-3-en-1-yl)-7-oxo-6,7-dihydro-1*H*-pyrrolo[2,3-*c*]pyridin-4-yl)-1-methyl-1*H*-benzo[*d*]imidazole-6-carboxylic acid** (**I–4**). To a solution of intermediate **I-1** (400 mg, 0.95 mmol, 1.0 equiv) and intermediate **I-2** (313 mg, 1.425 mmol, 1.5 equiv) in dioxane/H_2_O (6 mL, 5/1) were added Cs_2_CO_3_ (618 mg, 1.90 mmol, 2.0 equiv) and PdCl_2_(dppf) (69 mg, 0.095 mmol, 0.1 equiv), then the mixture was heated at 100 ^o^C under N_2_. After 3 h, the reaction mixture was concentrated in vacuo, and the residue was extracted with EA (40 mL* 3) and washed with brine (40 mL* 3). The combined organic layers were concentrated in vacuo and the residue was purified by flash chromatography (DCM/MeOH = 99% to 25%) to afford the desired intermediate **I-3** as black solid (410 mg, 84% yield). HRMS (ESI-TOF) m/z: [M + H]^+^ calcd for C_27_H_25_N_4_O_5_S, 517.1540; found, 517.1528.

To a solution of last step desired intermediate **I – 3** (275 mg, 0.63 mmol, 1.0 equiv) in DMF (2 mL) were added K_2_CO_3_ (146 mg, 1.06 mmol, 2 equiv) and MeI (50 µL, 0.636 mmol, 1.5 equiv). After being stirred at rt for 2 h, the resulting mixture was purified by reverse-ISCO to afford intermediate. To the obtained intermediate in MeOH/H_2_O (5:1, 3 mL) was added sodium hydroxide (NaOH, 106 mg, 2.65 mmol, 5 equiv). After being stirred at 80 ^o^C for 1 h, the resulting mixture was purified by reverse-ISCO to afford desired intermediate **I-4** as white solid (65 mg, 34% yield for two steps). ^1^H NMR (400 MHz, Methanol-*d*_4_) δ 9.09 (s, 1H), 8.49 (s, 1H), 8.30 (s, 1H), 7.54 (s, 1H), 7.46 – 7.34 (m, 1H), 6.29 (s, 1H), 5.96 – 5.76 (m, 1H), 5.12 – 4.99 (m, 2H), 4.28 – 4.03 (m, 5H), 2.69 – 2.50 (m, 2H).

**6-(But-3-en-1-yl)-4-(1-methyl-6-(piperazine-1-carbonyl)-1*H*-benzo[*d*]imidazol-4-yl)-1,6-dihydro-7*H*-pyrrolo[2,3-*c*]pyridin-7-one (I–5**). To a solution of **I - 4** (47 mg, 0.13 mmol, 1.0 equiv) in DMF (1 mL) were added *tert*-butyl piperazine-1-carboxylate (24.2 mg, 0.13 mmol, 1.0 equiv), *O*-(7-Azabenzotriazol-1-yl)-*N*,*N*,*N*',*N*'-tetramethyluronium hexafluorophosphate (HATU, 59 mg, 0.156 mmol, 1.2 equiv), and diisopropylethylamine (DIPEA, 66µL, 0.39 mmol, 3 equiv). The resulting mixture was stirred at room temperature for 30 min followed by purified by reverse-ISCO to afford the intermediate. To a solution of the obtained intermediate in DCM (1 mL) was added TFA (1 mL). The resulting mixture was stirred at rt for 30 min. Then all the volatiles were removed, the **I – 5** was obtained as a yellow solid (43 mg, 50% yield). ^1^H NMR (400 MHz, Methanol-*d*_4_) δ 9.25 (s, 1H), 8.04 (s, 1H), 7.77 (s, 1H), 7.50 (s, 1H), 7.38 (s, 1H), 6.26 (s, 1H), 5.95 – 5.79 (m, 1H), 5.15 – 4.98 (m, 2H), 4.23 – 4.13 (m, 5H), 4.06 – 3.79 (m, 4H), 3.42 – 3.31 (m, 4H), 2.63 – 2.49 (m, 2H).

**Scheme S2**. **General synthetic route for preparing compound 1-12***^a^*


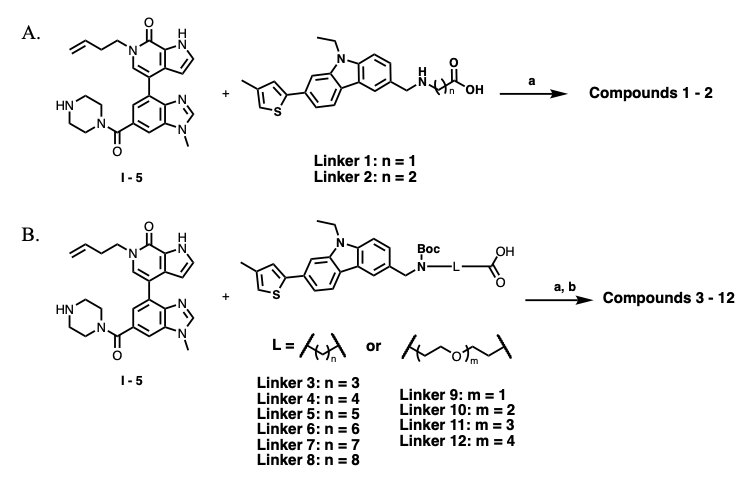


*^a^*Reaction and conditions: (a) HATU, DIPEA, DMF, rt; (b) HCl, MeOH, rt, 30 min.

**6-(But-3-en-1-yl)-4-(6-(4-(((9-ethyl-7-(4-methylthiophen-2-yl)-9*H*-carbazol-3-yl)methyl)glycyl)piperazine-1-carbonyl)-1-methyl-1*H*-benzo[*d*]imidazol-4-yl)-1,6-dihydro-7*H*-pyrrolo[2,3-*c*]pyridin-7-one** (**1**). To a solution of **linker 1** (3.7 mg, 0.0075 mmol, 1.0 equiv) in DMF (0.5 mL) were added **I - 5** (5 mg, 0.0075 mmol, 1.0 equiv), *O*-(7-Azabenzotriazol-1-yl)-*N*,*N*,*N*',*N*'-tetramethyluronium hexafluorophosphate (HATU, 3.4 mg, 0.009 mmol, 1.2 equiv), and diisopropylethylamine (DIPEA, 4 µL, 0.0225 mmol, 3 equiv). The resulting mixture was stirred at room temperature for 30 min followed by purified by preparative HPLC to afford compound **1** as a white solid (3.9 mg, 51% yield). ^1^H NMR (400 MHz, Methanol-*d*_4_) δ 9.11 (s, 1H), 8.21 (s, 1H), 8.09 (d, *J* = 8.0 Hz, 1H), 7.89 (s, 1H), 7.76 – 7.69 (m, 1H), 7.65 (s, 1H), 7.58 (q, *J* = 8.6 Hz, 2H), 7.53 – 7.44 (m, 2H), 7.38 (d, *J* = 2.8 Hz, 1H), 7.32 (s, 1H), 6.95 (s, 1H), 6.27 (d, *J* = 2.9 Hz, 1H), 5.88 (td, *J* = 17.0, 7.1 Hz, 1H), 5.05 (dd, *J* = 24.3, 13.6 Hz, 2H), 4.57 – 4.38 (m, 4H), 4.21 (t, *J* = 7.4 Hz, 2H), 4.11 (s, 5H), 3.56 (dd, *J* = 51.4, 32.0 Hz, 8H), 2.58 (q, *J* = 7.3 Hz, 2H), 2.29 (s, 3H), 1.41 (t, *J* = 7.1 Hz, 3H). HRMS (ESI-TOF) m/z: [M + H]^+^ calcd for C_46_H_47_N_8_O_3_S, 791.3486; found, 791.3475.

**6-(But-3-en-1-yl)-4-(6-(4-(3-(((9-ethyl-7-(4-methylthiophen-2-yl)-9*H*-carbazol-3-yl)methyl)amino)propanoyl)piperazine-1-carbonyl)-1-methyl-1*H*-benzo[*d*]imidazol-4-yl)-1,6-dihydro-7*H*-pyrrolo[2,3-*c*]pyridin-7-one** (**2**). Compound **2** was synthesized following the standard procedure for preparing compound **1** from **linker 2** and **I-5**. White solid, 2.9 mg, 37% yield. ^1^H NMR (400 MHz, Methanol-*d*_4_) δ 9.45 (s, 1H), 8.24 (s, 1H), 8.13 – 7.98 (m, 2H), 7.80 – 7.68 (m, 2H), 7.62 – 7.56 (m, 2H), 7.53 – 7.46 (m, 2H), 7.40 (s, 1H), 7.34 (s, 1H), 6.96 (s, 1H), 6.25 (s, 1H), 5.96 – 5.76 (m, 1H), 5.14 – 4.99 (m, 2H), 4.53 – 4.40 (m, 4H), 4.23 – 4.16 (m, 4H), 3.85 – 3.49 (m, 9H), 2.97 – 2.83 (m, 2H), 2.66 – 2.52 (m, 2H), 2.30 (s, 3H), 1.46 – 1.39 (m, 3H), 1.29 – 1.26 (m, 2H). ^13^C NMR (101 MHz, Methanol-*d*_4_) δ 171.00, 170.63, 156.13, 145.98, 144.72, 142.31, 139.99, 139.84, 135.76, 135.18, 134.61, 134.23, 132.47, 131.04, 130.47, 129.44, 128.44, 127.31, 126.77, 126.59, 124.81, 124.56, 123.18, 123.01, 122.64, 121.85, 121.25, 118.82, 117.99, 112.10, 111.67, 110.52, 106.71, 103.31, 52.87, 44.00, 38.50, 35.10, 34.00, 29.87, 17.54, 15.86, 15.81, 14.07. HRMS (ESI-TOF) m/z: [M + H]^+^ calcd for C_47_H_49_N_8_O_3_S, 805.3643; found, 805.3630.

**6-(But-3-en-1-yl)-4-(6-(4-(4-(((9-ethyl-7-(4-methylthiophen-2-yl)-9*H*-carbazol-3-yl)methyl)amino)butanoyl)piperazine-1-carbonyl)-1-methyl-1*H*-benzo[*d*]imidazol-4-yl)-1,6-dihydro-7*H*-pyrrolo[2,3-*c*]pyridin-7-one** (**3**). To a solution of **linker 3** (5.1 mg, 0.01 mmol, 1.0 equiv.) were added **I-5** (6.6 mg, 0.01 mmol, 1.0 equiv) *O*-(7-Azabenzotriazol-1-yl)-*N*,*N*,*N*',*N*'-tetramethyluronium hexafluorophosphate (HATU, 4.6 mg, 0.012 mmol, 1.2 equiv), and diisopropylethylamine (DIPEA, 5 µL, 0.03 mmol, 3 equiv). The resulting mixture was stirred at room temperature for 30 min followed by purified by preparative HPLC to afford intermediate. The obtained product was dissolved in MeOH (0.5 mL) followed by HCl (4M in dioxane, 0.5 mL). The resulting mixture was stirred at room temperature for 0.5 h. Then all the volatiles were removed, then **3** was obtained as a white solid (7.8 mg, 87% yield).^1^H NMR (400 MHz, Methanol-*d*_4_) δ 9.59 (s, 1H), 8.24 (s, 1H), 8.18 – 8.02 (m, 2H), 7.87 – 7.70 (m, 2H), 7.63 – 7.34 (m, 6H), 6.94 (s, 1H), 6.26 (s, 1H), 5.99 – 5.86 (m, 1H), 5.14 – 5.04 (m, 2H), 4.55 – 4.39 (m, 4H), 4.33 – 4.21 (m, 4H), 3.75 – 3.55 (m, 9H), 3.22 – 3.08 (m, 2H), 2.77 – 2.56 (m, 4H), 2.29 (s, 3H), 2.12 – 2.00 (m, 2H), 1.41 (s, 3H). HRMS (ESI-TOF) m/z: [M + H]^+^ calcd for C_48_H_51_N_8_O_3_S, 819.3799; found, 819.3778.

**6-(But-3-en-1-yl)-4-(6-(4-(5-(((9-ethyl-7-(4-methylthiophen-2-yl)-9*H*-carbazol-3-yl)methyl)amino)pentanoyl)piperazine-1-carbonyl)-1-methyl-1*H*-benzo[*d*]imidazol-4-yl)-1,6-dihydro-7*H*-pyrrolo[2,3-*c*]pyridin-7-one** (**4**). Compound **4** was synthesized following the standard procedure for preparing compound **3** from **linker 4** and **I-5**. White solid, 9.7 mg, 39% yield. ^1^H NMR (400 MHz, Methanol-*d*_4_) δ 9.56 (s, 1H), 8.24 (s, 1H), 8.18 – 7.95 (m, 2H), 7.85 – 7.67 (m, 2H), 7.66 – 7.38 (m, 5H), 7.32 (s, 1H), 6.95 (s, 1H), 6.25 (s, 1H), 5.99 – 5.83 (m, 1H), 5.12 – 5.01 (m, 2H), 4.52 – 4.33 (m, 4H), 4.29 – 4.16 (m, 4H), 3.75 – 3.57 (m, 9H), 3.19 – 2.99 (m, 2H), 2.70 – 2.44 (m, 4H), 2.29 (s, 3H), 1.87 – 1.69 (m, 4H), 1.40 (s, 3H). HRMS (ESI-TOF) m/z: [M + H]^+^ calcd for C_49_H_53_N_8_O_3_S, 833.3956; found, 833.3967.

**6-(But-3-en-1-yl)-4-(6-(4-(6-(((9-ethyl-7-(4-methylthiophen-2-yl)-9*H*-carbazol-3-yl)methyl)amino)hexanoyl)piperazine-1-carbonyl)-1-methyl-1*H*-benzo[*d*]imidazol-4-yl)-1,6-dihydro-7*H*-pyrrolo[2,3-*c*]pyridin-7-one** (**5**). Compound **5** was synthesized following the standard procedure for preparing compound **3** from **linker 5** and **I-5**. White solid, 7.6 mg, 83% yield. ^1^H NMR (400 MHz, Methanol-*d*_4_) δ 9.60 (s, 1H), 8.24 (s, 1H), 8.19 – 7.95 (m, 2H), 7.84 – 7.67 (m, 2H), 7.63 – 7.20 (m, 6H), 6.94 (s, 1H), 6.25 (s, 1H), 6.08 – 5.85 (m, 1H), 5.12 – 5.02 (m, 2H), 4.53 – 4.33 (m, 4H), 4.30 – 4.04 (m, 4H), 3.89 – 3.50 (m, 9H), 3.20 – 3.00 (m, 2H), 2.76 – 2.37 (m, 4H), 2.29 (s, 3H), 1.90 – 1.63 (m, 4H), 1.53 – 1.32 (m, 5H). HRMS (ESI-TOF) m/z: [M + H]^+^ calcd for C_50_H_55_N_8_O_3_S, 847.4112; found, 847.4141.

**6-(But-3-en-1-yl)-4-(6-(4-(7-(((9-ethyl-7-(4-methylthiophen-2-yl)-9*H*-carbazol-3-yl)methyl)amino)heptanoyl)piperazine-1-carbonyl)-1-methyl-1*H*-benzo[*d*]imidazol-4-yl)-1,6-dihydro-7*H*-pyrrolo[2,3-*c*]pyridin-7-one** (**6**). Compound **6** was synthesized following the standard procedure for preparing compound **3** from **linker 6** and **I-5**. White solid, 6.6 mg, 71% yield. ^1^H NMR (400 MHz, Methanol-*d*_4_) δ 9.58 (s, 1H), 8.34 – 8.03 (m, 3H), 7.85 – 7.70 (m, 2H), 7.60 – 7.33 (m, 6H), 6.94 (s, 1H), 6.26 (s, 1H), 6.08 – 5.81 (m, 1H), 5.12 – 5.00 (m, 2H), 4.47 – 4.34 (m, 4H), 4.31 – 4.21 (m, 4H), 3.76 – 3.58 (m, 9H), 3.15 – 2.99 (m, 2H), 2.63 – 2.42 (m, 4H), 2.29 (s, 3H), 1.75 – 1.58 (m, 4H), 1.47 – 1.37 (m, 7H). HRMS (ESI-TOF) m/z: [M + H]^+^ calcd for C_51_H_57_N_8_O_3_S, 861.4269; found, 861.4255.

**6-(But-3-en-1-yl)-4-(6-(4-(8-(((9-ethyl-7-(4-methylthiophen-2-yl)-9*H*-carbazol-3-yl)methyl)amino)octanoyl)piperazine-1-carbonyl)-1-methyl-1*H*-benzo[*d*]imidazol-4-yl)-1,6-dihydro-7*H*-pyrrolo[2,3-*c*]pyridin-7-one** (**7**). Compound **7** was synthesized following the standard procedure for preparing compound **3** from **linker 7** and **I-5**. White solid, 8.5 mg, 90% yield. ^1^H NMR (400 MHz, Methanol-*d*_4_) δ 9.56 (s, 1H), 8.22 (s, 1H), 8.14 – 7.93 (m, 2H), 7.85 – 7.67 (m, 2H), 7.66 – 7.46 (m, 4H), 7.46 – 7.33 (m, 2H), 6.94 (s, 1H), 6.25 (s, 1H), 5.98 – 5.79 (m, 1H), 5.14 – 5.02 (m, 2H), 4.51 – 4.34 (m, 4H), 4.27 – 4.15 (m, 4H), 3.79 – 3.59 (m, 9H), 3.12 – 2.97 (m, 2H), 2.68 – 2.52 (m, 2H), 2.49 – 2.34 (m, 2H), 2.29 (s, 3H), 1.75 – 1.67 (m, 2H), 1.64 – 1.54 (m, 2H), 1.44 – 1.34 (m, 9H). HRMS (ESI-TOF) m/z: [M + H]^+^ calcd for C_52_H_59_N_8_O_3_S, 875.4425; found, 875.4428.

**6-(But-3-en-1-yl)-4-(6-(4-(9-(((9-ethyl-7-(4-methylthiophen-2-yl)-9*H*-carbazol-3-yl)methyl)amino)nonanoyl)piperazine-1-carbonyl)-1-methyl-1*H*-benzo[*d*]imidazol-4-yl)-1,6-dihydro-7*H*-pyrrolo[2,3-*c*]pyridin-7-one** (**8**). Compound **8** was synthesized following the standard procedure for preparing compound **3** from **linker 8** and **I-5**. White solid, 8.0 mg, 83% yield. ^1^H NMR (400 MHz, Methanol-*d*_4_) δ 9.62 (s, 1H), 8.27 – 7.98 (m, 3H), 7.80 – 7.66 (m, 2H), 7.59 – 7.32 (m, 6H), 6.94 (s, 1H), 6.27 (s, 1H), 6.08 – 5.74 (m, 1H), 5.22 – 5.06 (m, 2H), 4.53 – 4.39 (m, 4H), 4.35 – 4.21 (m, 4H), 3.80 – 3.50 (m, 9H), 3.20 – 3.03 (m, 2H), 2.68 – 2.54 (m, 2H), 2.51 – 2.36 (m, 2H), 2.29 (s, 3H), 1.77 – 1.59 (m, 4H), 1.49 – 1.32 (m, 11H). HRMS (ESI-TOF) m/z: [M + H]^+^ calcd for C_53_H_61_N_8_O_3_S, 889.4582; found, 889.4576.

**6-(Bbut-3-en-1-yl)-4-(6-(4-(3-(2-(((9-ethyl-7-(4-methylthiophen-2-yl)-9*H*-carbazol-3-yl)methyl)amino)ethoxy)propanoyl)piperazine-1-carbonyl)-1-methyl-1*H*-benzo[*d*]imidazol-4-yl)-1,6-dihydro-7*H*-pyrrolo[2,3-*c*]pyridin-7-one** (**9**). Compound **9** was synthesized following the standard procedure for preparing compound **3** from **linker 9** and **I-5**. White solid, 9.8 mg, 81% yield. ^1^H NMR (400 MHz, Methanol-*d*_4_) δ 9.53 (s, 1H), 8.34 – 7.97 (m, 3H), 7.83 – 7.54 (m, 5H), 7.43 – 7.25 (m, 3H), 6.93 (s, 1H), 6.20 (s, 1H), 6.00 – 5.74 (m, 1H), 5.12 – 5.01 (m, 2H), 4.49 – 4.35 (m, 4H), 4.30 – 4.16 (m, 4H), 3.79 – 3.62 (m, 15H), 2.82 – 2.68 (m, 2H), 2.64 – 2.51 (m, 2H), 2.28 (s, 3H), 1.43 – 1.30 (m, 3H). HRMS (ESI-TOF) m/z: [M + H]^+^ calcd for C_49_H_53_N_8_O_4_S, 849.3905; found, 849.3914.

**6-(But-3-en-1-yl)-4-(6-(4-(3-(2-(2-(((9-ethyl-7-(4-methylthiophen-2-yl)-9*H*-carbazol-3-yl)methyl)amino)ethoxy)ethoxy)propanoyl)piperazine-1-carbonyl)-1-methyl-1*H*-benzo[*d*]imidazol-4-yl)-1,6-dihydro-7*H*-pyrrolo[2,3-*c*]pyridin-7-one** (**10**). Compound **10** was synthesized following the standard procedure for preparing compound **3** from **linker 10** and **I-5**. White solid, 6.8 mg, 59% yield. ^1^H NMR (400 MHz, Methanol-*d*_4_) δ 9.57 (s, 1H), 8.31 – 8.02 (m, 3H), 7.70 – 7.52 (m, 5H), 7.46 – 7.31 (m, 3H), 6.90 (s, 1H), 6.23 (s, 1H), 6.01 – 5.71 (m, 1H), 5.07 – 5.00 (m, 2H), 4.50 – 4.42 (m, 4H), 4.29 – 4.18 (m, 4H), 3.73 – 3.55 (m, 19H), 2.70 – 2.55 (m, 4H), 2.26 (s, 3H), 1.45 – 1.39 (m, 3H). HRMS (ESI-TOF) m/z: [M + H]^+^ calcd for C_51_H_57_N_8_O_5_S, 893.4167; found, 893.4195.

**6-(But-3-en-1-yl)-4-(6-(4-(1-(9-ethyl-7-(4-methylthiophen-2-yl)-9*H*-carbazol-3-yl)-5,8,11-trioxa-2-azatetradecan-14-oyl)piperazine-1-carbonyl)-1-methyl-1*H*-benzo[*d*]imidazol-4-yl)-1,6-dihydro-7*H*-pyrrolo[2,3-*c*]pyridin-7-one** (**11**). Compound **11** was synthesized following the standard procedure for preparing compound **3** from **linker 11** and **I-5**. White solid, 8.5 mg, 70% yield. ^1^H NMR (400 MHz, Methanol-*d*_4_) δ 9.58 (s, 1H), 8.25 (s, 1H), 8.16 – 7.94 (m, 2H), 7.78 – 7.56 (m, 4H), 7.52 – 7.25 (m, 4H), 6.90 (s, 1H), 6.25 (s, 1H), 5.99 – 5.72 (m, 1H), 5.08 – 5.00 (m, 2H), 4.51 – 4.38 (m, 4H), 4.29 – 4.15 (m, 4H), 3.80 – 3.53 (m, 23H), 2.74 – 2.46 (m, 4H), 2.26 (s, 3H), 1.45 – 1.37 (m, 3H). HRMS (ESI-TOF) m/z: [M + H]^+^ calcd for C_53_H_61_N_8_O_6_S, 937.4429; found, 937.4445.

**6-(But-3-en-1-yl)-4-(6-(4-(1-(9-ethyl-7-(4-methylthiophen-2-yl)-9*H*-carbazol-3-yl)-5,8,11,14-tetraoxa-2-azaheptadecan-17-oyl)piperazine-1-carbonyl)-1-methyl-1*H*-benzo[*d*]imidazol-4-yl)-1,6-dihydro-7*H*-pyrrolo[2,3-*c*]pyridin-7-one** (**12**). Compound **12** was synthesized following the standard procedure for preparing compound **3** from **linker 12** and **I-5**. White solid, 10.1 mg, 80% yield. ^1^H NMR (400 MHz, Methanol-*d*_4_) δ 9.55 (s, 1H), 8.24 (s, 1H), 8.16 – 7.98 (m, 2H), 7.78 – 7.67 (m, 2H), 7.61 – 7.40 (m, 5H), 7.32 (s, 1H), 6.92 (s, 1H), 6.24 (s, 1H), 6.06 – 5.70 (m, 1H), 5.21 – 5.02 (m, 2H), 4.53 – 4.39 (m, 4H), 4.26 – 4.18 (m, 4H), 3.77 – 3.50 (m, 27H), 2.66 – 2.50 (m, 4H), 2.27 (s, 3H), 1.44 – 1.38 (m, 3H). HRMS (ESI-TOF) m/z: [M + H]^+^ calcd for C_55_H_65_N_8_O_7_S, 981.4691; found, 981.4687.

**References:**

[1] X. Hu, M. Kabir, Y. Lin, Y. Xiong, R. E. Parsons, W. Gu, J. Jin, *J. Med. Chem.* **2024**, *67*, 14633-14648.

[2] S. Wang, V. Tsui, T. D. Crawford, J. E. Audia, D. J. Burdick, M. H. Beresini, A. Cote, R. Cummings, M. Duplessis, E. M. Flynn, M. C. Hewitt, H. R. Huang, H. Jayaram, Y. Jiang, S. Joshi, J. Murray, C. G. Nasveschuk, E. Pardo, F. Poy, F. A. Romero, Y. Tang, A. M. Taylor, J. Wang, Z. Xu, L. E. Zawadzke, X. Zhu, B. K. Albrecht, S. R. Magnuson, S. Bellon, A. G. Cochran, *J. Med. Chem.* **2018**, *61*, 9301-9315.

**^1^H NMR spectrum of MS172**

**^13^C NMR spectrum of MS172**

**LC-MS spectrum of MS172**
